# An Insight into the Chromosomal Evolution of Lebiasinidae (Teleostei, Characiformes)

**DOI:** 10.3390/genes11040365

**Published:** 2020-03-28

**Authors:** Francisco de M. C. Sassi, Terumi Hatanaka, Renata Luiza R. de Moraes, Gustavo A. Toma, Ezequiel A. de Oliveira, Thomas Liehr, Petr Rab, Luiz A. C. Bertollo, Patrik F. Viana, Eliana Feldberg, Mauro Nirchio, Manoela Maria F. Marinho, José Francisco de S. e Souza, Marcelo de B. Cioffi

**Affiliations:** 1 Laboratório de Citogenética de Peixes, Departamento de Genética e Evolução, Universidade Federal de São, Carlos, SP 13565-905, Brazil; fmcsassi@estudante.ufscar.br (F.d.M.C.S.); hterumi@yahoo.com.br (T.H.); rlrdm@hotmail.com (R.L.R.d.M.); gustavo_toma@hotmail.com (G.A.T.); bertollo@ufscar.br (L.A.C.B.); mbcioffi@ufscar.br (M.d.B.C.); 2 Secretaria de Estado de Educação do Mato Grosso–SEDUC-MT, Cuiabá, MT 78049-909, Brazil; ezekbio@gmail.com; 3 Institute of Human Genetics, University Hospital Jena, Jena 07747, Germany; 4 Laboratory of Fish Genetics, Institute of Animal Physiology and Genetics, Czech Academy of Sciences, 27721 Liběchov, Czech Republic; Rab@iapg.cas.cz; 5 Laboratório de Genética Animal, Coordenação de Biodiversidade, Instituto Nacional de Pesquisas da Amazônia, Manaus, AM 69067-375, Brazil; patrik.biologia@gmail.com (P.F.V.); feldberg@inpa.gov.br (E.F.); sousa.josef@gmail.com (J.F.d.S.e.S.); 6 Facultad de Ciencias Agropecuarias, Universidad Técnica de Machala, Machala 070151, Ecuador; mauro.nirchio@gmail.com; 7 Museu de Zoologia da Universidade de São Paulo (MZUSP), São Paulo, SP 04263-000, Brazil; manumfm@yahoo.com.br; 8 Departamento de Sistemática e Ecologia, Universidade Federal da Paraíba, João Pessoa, PB 58033-455, Brazil

**Keywords:** comparative genomic hybridization, ribosomal DNA, Neotropical fishes, cytogenetics, karyotype

## Abstract

Lebiasinidae fishes have been historically neglected by cytogenetical studies. Here we present a genomic comparison in eleven Lebiasinidae species, in addition to a review of the ribosomal DNA sequences distribution in this family. With that, we develop ten sets of experiments in order to hybridize the genomic DNA of representative species from the genus *Copeina*, *Copella*, *Nannostomus,* and *Pyrrhulina* in metaphase plates of *Lebiasina melanoguttata*. Two major pathways on the chromosomal evolution of these species can be recognized: (i) conservation of 2n = 36 bi-armed chromosomes in Lebiasininae, as a basal condition, and (ii) high numeric and structural chromosomal rearrangements in Pyrrhulininae, with a notable tendency towards acrocentrization. The ribosomal DNA (rDNA) distribution also revealed a marked differentiation during the chromosomal evolution of Lebiasinidae, since both single and multiple sites, in addition to a wide range of chromosomal locations can be found. With some few exceptions, the terminal position of 18S rDNA appears as a common feature in Lebiasinidae-analyzed species. Altogether with Ctenoluciidae, this pattern can be considered a symplesiomorphism for both families. In addition to the specific repetitive DNA content that characterizes the genome of each particular species, *Lebiasina* also keeps inter-specific repetitive sequences, thus reinforcing its proposed basal condition in Lebiasinidae.

## 1. Introduction

Advanced molecular approaches have been widely applied in cytogenetic studies of many animal groups, providing useful insights into their karyotype differentiation and genome evolution. Although in fishes, such procedures have also improved investigations as a whole, chromosomal analysis of several taxa is still emerging [1]. Obtaining good metaphases plates, both in quantity and quality, stands out as the reason for such scenarios, mainly for small-sized and miniature fishes. Thus, dealing with chromosomes of miniature species, which reach a maximum length of 26 mm in maturity [2], is challenging, but possible [3,4,5,6,7].

Lebiasinidae is a freshwater characiform family comprising about 75 recognized species [8], distributed throughout Central and South America except Chile, which experienced body miniaturization along with their evolution [2]. Two distinguishable subfamilies are recognized: (i) Lebiasininae, comprising *Lebiasina, Piabucina* and *Derhamia,* and (ii) Pyrrhulininae, including *Copeina, Copella, Nannostomus,* and *Pyrrhulina* [8]. However, Netto-Ferreira [9] proposed the inclusion of *Derhamia* in Pyrrhulininae, based on morphological characters.

The phylogenetic position of Lebiasinidae within the order Characiformes has been frequently discussed [10,11,12,13,14], but without a conclusive solution. Recent analyses based on molecular data showed that Ctenoluciidae emerged as the sister group of Lebiasinidae [15,16,17]. A further indication of such close relationship was found using cytogenetic approaches where whole chromosome painting (WCP) experiments with probes from the first chromosome pair of *Lebiasina bimaculata* (Lebiasinidae) and *Boulengerella lateristriga* (Ctenoluciidae) revealed great similarity between them; a fact also extended to other Ctenoluciidae species [6]. Additionally, a comparative genomic hybridization (CGH) experiment showed co-localized scattered signals on *L. bimaculata* and *B. lateristriga* chromosomes, indicating that shared syntenic regions remained conserved during the evolutionary process of these groups [6].

*Lebiasina* (Lebiasininae) is one of the most unexplored taxa among Lebiasinidae in terms of cytogenetic data. It is considered a basal group within Lebiasinidae, with morphological [9,18] and cytogenetic [6] features corroborating such position. This makes *Lebiasina* an interesting group for evolutionary studies. For such purposes, CGH is a helpful methodology that has improved the evolutionary cytogenetics field by comparing entire genomes. Although initially developed to use in clinical approaches [19], CGH is now successfully used to trace evolutionary trends among different metazoan groups. In fishes, distinctive evolutionary processes (including the differentiation of sex chromosomes) have been highlighted among different species and groups using this advanced technique [5,6,20,21,22]. 

This study is part of a series focusing on the chromosomal evolution of the Lebiasinidae. Here, CGH experiments were used for the cross-species painting of 11 lebiasinid species and to revise the distribution of ribosomal sequences across their genomes, thus providing additional insight into their chromosomal evolution.

## 2. Materials and Methods 

### 2.1. Samples

**Figure 1:**
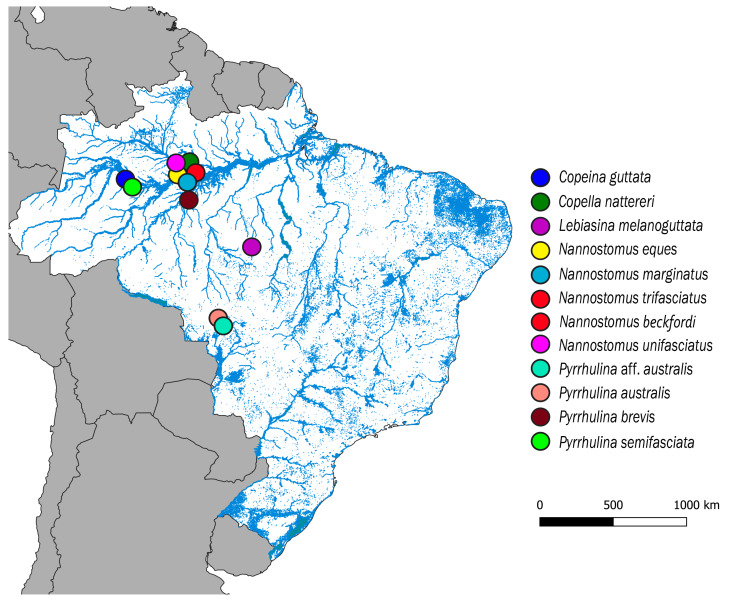
Map of the central portion of South America showing the Brazilian sample sites of Copeina guttata, Copella nattereri, Lebiasina melanoguttata, Nannostomus eques, N. marginatus, N. trifasciatus, N. unifasciatus, Pyrrhulina australis, Pyrrhulina aff. australis, P. brevis and P. semifasciata. The map was produced using the software QGis 3.4.4 (https://qgis.org), Inkscape 0.92 (https://inkscape.org), and Adobe Photoshop CC 2015 (San Jose, CA, USA).

Eleven Lebiasinidae species from several Brazilian rivers were analyzed (Figure 1; Table 1). Fieldwork had authorization from Brazilian Environmental Agencies ICMBIO/SISBIO (License number 48628-2) and SISGEN (A96FF09). Individuals were taxonomically identified and deposited at the Museu de Zoologia da Universidade de São Paulo (MZUSP; Table 1)

### 2.2. Chromosome Preparations and Ideograms 

Mitotic chromosomes were prepared by the direct conventional air-drying technique [23] from kidney cells. All experiments followed the ethical/anesthesia conducts and were approved by the Ethics Committee on Animal Experimentation of the Universidade Federal de São Carlos (Process number CEUA 1853260315). Schematic representations, to demonstrate the chromosomal distribution of the 5S and 18S rDNA sequences in respective representative Ctenoluciidae and Lebiasinidae, were arranged using the Adobe Photoshop CC 2015 (San Jose, CA, USA), according to the data from [3,4,5,6,7,24]. Four genera were not included in our ideogram since there is no available data for the rDNA position on chromosomes of *Copella*, *Derhamia, Piabucina* (Lebiasinidae), and *Ctenolucius* (Ctenoluciidae).

### 2.3. Probes for Comparative Genomic Hybridization (CGH)

Ten sets of experiments were undertaken to hybridize the genomic DNA (gDNA) of *Copeina*, *Copella*, *Nannostomus,* and *Pyrrhulina* species under study onto metaphase plates of *Lebiasina melanoguttata*. For this purpose, the female-derived gDNA of *L. melanoguttata*, *C. guttata*, *C. nattereri*, *P. australis*, *Pyrrhulina* aff. *australis*, *P. brevis*, *P. semifasciata*, *N. eques*, *N. marginatus*, *N. trifasciatus,* and *N. unifasciatus* were extracted from liver tissues by a standard phenol–chloroform–isoamyl alcohol method [25]. For all assays, the female-derived gDNA of *L. melanoguttata* was directly labeled with Atto488 (green fluorescence) using the Nick-translation labeling kit (Jena Bioscience, Jena, Germany), while the gDNA of *C. guttata*, *C. nattereri*, *P. australis*, *Pyrrhulina* aff. *australis*, *P. brevis*, *P. semifasciata*, *N. eques*, *N. marginatus*, *N. trifasciatus,* and *N. unifasciatus* were directly labeled with Atto550 (red fluorescence) also using the Nick-translation labeling kit (Jena Bioscience, Jena, Germany). The final hybridization mixtures contained 500 ng of *L. melanoguttata* gDNA plus 500 ng of gDNA from one of the above-described species. In all experiments, repetitive sequences were blocked using 15 μg of C0t-1 female-derived DNA from each species, prepared according to Zwick et al. [26], and dissolved in 20 μL of the hybridization buffer (50% formamide, 2x SSC, 10% SDS, 10% dextran sulfate, and Denhardt’s buffer, pH 7.0). The chosen ratio of probe vs. C0t-1 DNA amount was based on the experiments performed in previous studies in several fish groups [5,6,20,27].

### 2.4. Fluorescence in Situ Hybridization (FISH) for CGH

CGH experiments were performed using the protocol of Symonová et al. [27]. Slides were first aged for 1 to 2 h at 60 °C and then treated with RNase A (20 μg/mL; 90 min at 37 °C in a wet chamber), and pepsin (50 μg/mL; 3 min at 37 °C). Chromosomes were denatured in 75% formamide diluted in 2x SSC at 74 °C for 3 min. At the same time, the probes were also denatured at 86 °C for 10 min and chilled on ice for 10 min. Then, the hybridization mix was applied to the slides, followed by a three-day incubation in a wet chamber (37 °C). The non-specific hybridization remnants were removed by a stringent washing at 44 °C, two washes in 50% formamide/2x SSC (10 min each), three washes in 1x SSC (7 min each), and a final wash in 2x SSC at room temperature. Chromosomes were counterstained with DAPI (1.2 μg/mL) and mounted in an antifade solution (Vector, Burlingame, CA, USA).

## 3. Results

### 3.1. Chromosomal Distribution of the rDNA Sequences Across the Genome of Lebiasinidae and Ctenoluciidae Species Understudy

**Figure 2:**
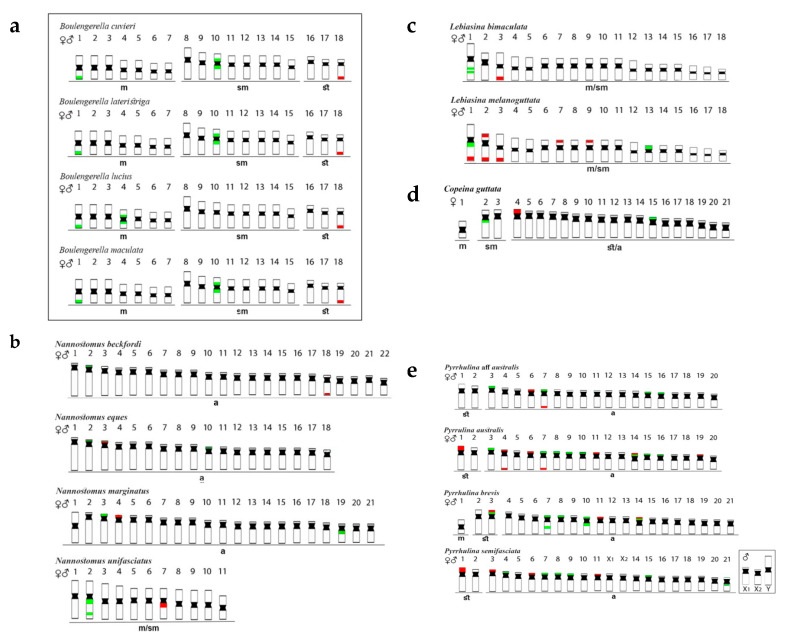
Schematic representation of chromosomes of Lebiasinidae and Ctenoluciidae species, highlighting the position of 5S rDNA (green) and 18S rDNA (red). The small box highlights a sex chromosome system in *Pyrrhulina semifasciata*, while the bigger box highlights the Ctenoluciidae members. FISH data were taken from [3,4,5,6,7,24]. Letters correspond to the investigated genera: (**a**)—*Boulengerella*, (**b**)—*Nannostomus*, (**c**)—*Lebiasina*, (**d**)—*Copeina*, and (**e**)—*Pyrrhulina*.

*Boulengerella* (Ctenoluciidae) species (Figure 2a) had 5S rDNA sites located in the terminal and pericentromeric regions of the first and the 10th chromosome pairs, respectively. The only exception for this pattern occurred in *B. lucius*, which had the fourth chromosome pair, instead of the tenth one, bearing these sites. As to the 18S rDNA, it was found only in the telomeric region of the 18th pair in the karyotypes of all *Boulengerella* species [4].

*Nannostomus* species (Figure 2b) possessed 5S rDNA sequences in one chromosome pair only, although with variable positions, i.e., (1) telomeric region of the short (p) arms of the pairs 03 of *N. eques* and 04 of *N. marginatus*, (2) proximal region of the long (q) arms of the pair 07 of *N. unifasciatus*, and (3) telomeric region of the pair 18 of *N. beckfordi*. However, the 18S rDNA sites were more varied in distribution, both in number and location among species: (1) one signal in the telomeric region of the short arms of the 2nd chromosome pair of *N. beckfordi*, (2) two signals, both in the interstitial region of the q arms of the 2nd pair of *N. unifasciatus*, (3) one signal in the telomeric region of the p arms of the chromosomes 02 and 18 in *N. eques,* and (4) one telomeric signal in the p arms of the pair 03 of *N. marginatus*, with an additional pericentromeric signal in the q arms of pair 19 [24]. 

*Lebiasina* species (Figure 2c) also had distinct patterns of rDNA distribution. The Ecuadorian species *L. bimaculata* had 5S sites in the interstitial position of the first chromosome pair and 18S sites in the telomeric region of pair 03. On the other hand, the Brazilian species *L. melanoguttata* had multiple 18S sites, with 12 telomeric ones in the chromosome pairs 01, 02, 03, 07, and 09, but also including sites in both telomeric regions in pair 02. The 5S rDNA sequences were in the proximal region of the q arms of chromosome 01, with a probable paracentric inversion, together with the 18S rDNA, and also in the p arms of pair 13 [6].

*Copeina guttata* (Figure 2d) possessed 5S rDNA signals in the proximal region of the q arms of the second chromosome pair, and also in the short arms of the 15th one. On the other hand, the 18S rDNA has a single distribution, being located in the short arms of pair 04 [7].

*Pyrrhulina* species showed the most diversified rDNA distribution patterns than those of the other Lebiasinidae, with multiple 5S and 18S rDNA chromosomal sites (Figure 2e). In *P. semifasciata*, the p arms of the pairs 07, 08, 09, 15, and 21 possessed 5S rDNA sequences, while the 18S rDNA ones were located in the chromosomes 01, 03, 06, and 11. Similarly, *P. brevis* also had five chromosomes with 5S rDNA sequences in their short arms (pairs 03, 07, 08, 10, and 14). In the 7th pair, an additional interstitial signal occurs on the long arms, as well as in chromosome 10, but the proximal region. The 5S and 18S rDNA sequences were located in syntenic sites in p arms of chromosome pairs 03 and 14, in addition to pair 11 with 18S rDNA sites only. In *P. australis*, 18S rDNA sites were found in the p arms of the pairs 01, 06, 11, and 19, in both telomeric regions of pair 04, and also in this same region of the q arms of pair 07. The 5S and 18S sequences were in the p arms of pair 14 in the syntenic position, together with other 5S sites in the p arms of the chromosomes 03, 07, 08, 09, 10, 15, and 16. *Pyrrhulina* aff. *australis* possessed four chromosomes with 5S rDNA sites (pairs 03, 07, 15, and 16) in their p arms. 18S sequences were also in the 7th pair, but in the telomeric region of the q arms, besides an additional site in the p arms of pair 06 [3,5].

### 3.2. Comparative Genomic Hybridization (CGH)

**Figure 3:**
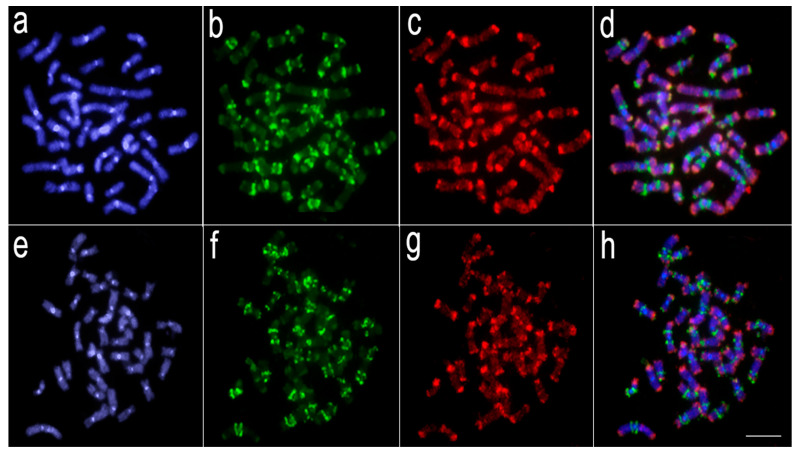
Comparative genomic hybridization using the gDNA of *Lebiasina melanoguttata*, *Copeina guttata,* and *Copella nattereri* against the chromosomal background of *Lebiasina melanoguttata*. Genomic probes from *L. melanoguttata* and *Copeina guttata* hybridized against *L. melanoguttata* chromosomes (**a**–**d**). Genomic probes from *L. melanoguttata* and *Copella nattereri* hybridized against *L. melanoguttata* chromosomes (**e**–**h**). The first column (**a**,**e**): DAPI images (blue); second column (**b**,**f**): hybridization patterns using gDNA probe from *L. melanoguttata*; third column (**c**,**g**): hybridization patterns using gDNA probes from *Copeina guttata* and *Copella nattereri*, respectively; fourth column (**d**,**h**) merged images of both genomic probes and DAPI staining depicting the common regions in yellow. Scale bar = 5 µm.

**Figure 4:**
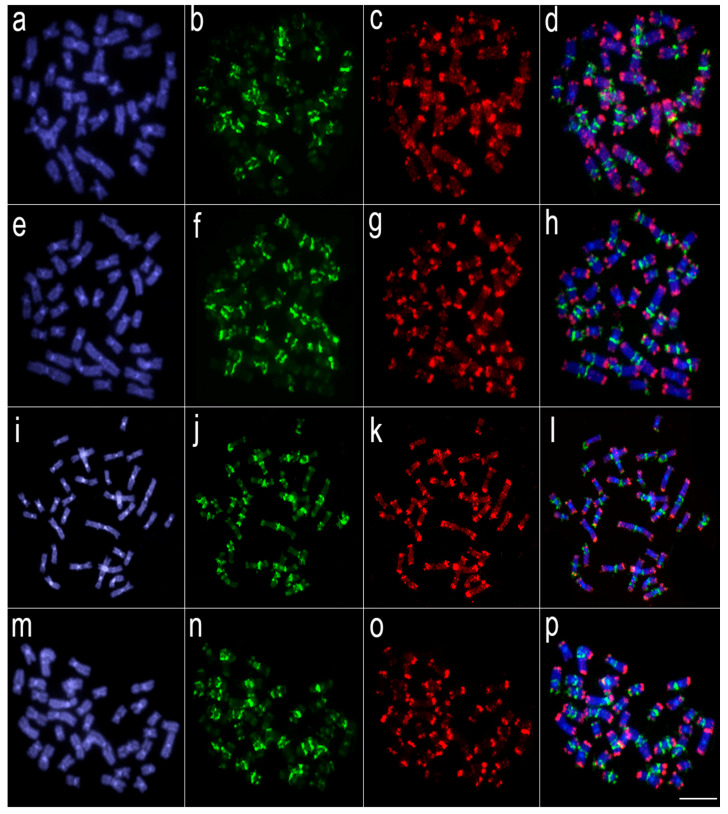
Comparative genomic hybridization using the gDNA of *Lebiasina melanoguttata* and *Pyrrhulina* species against a chromosomal background of *Lebiasina melanoguttata*. Genomic probes from *L. melanoguttata* and *P. australis* hybridized against *L. melanoguttata* chromosomes (**a**–**d**). Genomic probes from *L. melanoguttata* and *Pyrrhulina* aff. *australis* hybridized against *L. melanoguttata* chromosomes (**e**–**h**). Genomic probes from *L. melanoguttata* and *P. brevis* hybridized against *L. melanoguttata* chromosomes (**i**–**l**). Genome from *L. melanoguttata* and *P. semifasciata* hybridized against *L. melanoguttata* chromosomes (**m**–**p**). The first column (**a**,**e**,**I**,**m**): DAPI images (blue); second column (b, f, j, and n): hybridization patterns using gDNA probe from *L. melanoguttata*; third column (**c**,**g**,**k**,**o**): hybridization patterns using gDNA probes from *P. australis*, *Pyrrhulina* aff. *australis*, *P. brevis,* and *P. semifasciata*, respectively; fourth column (**d**,**h**,**l**,**p**) merged images of both genomic probes and DAPI staining, depicting the shared regions in yellow. Scale bar = 5 µm.

**Figure 5:**
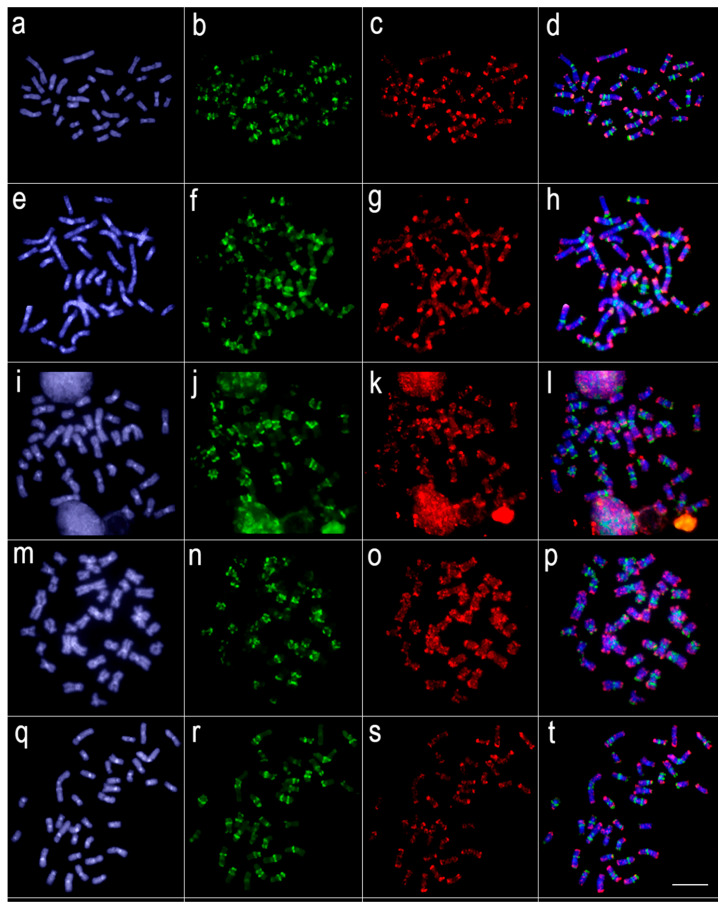
Comparative genomic hybridization among *Lebiasina melanoguttata* and *Nannostomus* species. Genomic probes from *L. melanoguttata* and *N. unifasciatus* hybridized against *L. melanoguttata* chromosomes (**a**–**d**). Genomic probes from *L. melanoguttata*, and *N. trifasciatus* hybridized against *L. melanoguttata* chromosomes (**e**–**h**). Genomic probes from *L. melanoguttata*, and *N. beckfordi* hybridized against *L. melanoguttata* chromosomes (**i**–**l**). Genomic probes from *L. melanoguttata* and *N. eques* hybridized against *L. melanoguttata* chromosomes (**m**–**p**). Genomic probes from *L. melanoguttata* and *N. marginatus* hybridized against *L. melanoguttata* chromosomes (**q**–**t**). The first column (**a**,**e**,**i**,**m**,**q**): DAPI images (blue); second column (**b**,**f**,**j**,**n**,**r**): hybridization patterns using gDNA probe from *L. melanoguttata*; third column (**c**,**g**,**k**,**o**,**s**): hybridization patterns using gDNA probes from *N. unifasciatus*, *N. trifasciatus*, *N. beckfordi*, *N. eques*, and *N. marginatus*, respectively; fourth column (**d**,**h**,**l**,**p**,**t**) merged images of both genomic probes and DAPI staining depicting the shared regions in yellow. Scale bar = 5 µm.

Comparative genomic hybridization (CGH) experiments revealed that a significant level of genomic divergence occurs between *L. melanoguttata* and the other lebiasinid species (Figure 3, Figure 4 and Figure 5). A high level of species-specific genomic compartmentalization stood out, with distinct patterns of repetitive DNA sequences both in amount and distribution in the chromosomes. Besides, some inter-specific segments of repetitive DNAs were also highlighted as shared among species.

## 4. Discussion

### Karyotype and Chromosomal Differentiation in Lebiasinidae 

Extensive chromosomal rearrangements, both in 2n and karyotype morphology, which may be probably linked to speciation processes, took place during the diversification of the Lebiasinidae. Overall, two major pathways can be recognized in the chromosomal evolution of the family: (i) conservation of 2n = 36 and karyotype composed of exclusively bi-armed chromosomes in the Lebiasininae as a basal condition; (ii) high 2n and structural chromosomal rearrangements in the Pyrrhulininae, with karyotypes prominently dominated by acrocentric chromosomes (Figure 2). These findings fit with the hypothesis that several derived fish clades predominantly possess mono-armed chromosomes, while basal ones have karyotypes dominated by bi-armed chromosomes [28].

Teleost fishes display varied modes of chromosomal evolution. It is noteworthy, for example, that several groups within Characiformes have more conserved karyotypes, maintaining the 2n very close or even equal to 54 and a relatively similar karyotype structure such as in Anostomidae, Curimatidae, Prochilodontidae, Hemiodontidae, and Chilodontidae fishes [29]. Such characteristics could be associated with the so-called karyotypic orthoselection [30], leading to the conservation of bi-armed chromosomes among related groups. However, rapid and recent speciation events can also create conserved karyotypes [31], a fact that cannot be ruled out for lebiasinid fishes since the only phylogenetic analysis of the family does not make references to divergence time [9]. Certainly, although Lebiasininae species possess a conserved karyotype macrostructure, interspecific genomic divergences are extensively observed, as here highlighted [6]. However, other fish groups show considerable divergences of the karyotype structure among its species, for example, the Erythrinidae [32,33], the Characidae in the Characiformes [29], and the Loricariidae in the Siluriformes [34,35,36]. Remarkably, both trends, i.e., (i) conservation of the basal condition 2n = 36 and karyotype composed exclusively by bi-armed chromosomes in Lebiasininae, and (ii) predominance of acrocentric chromosomes in the karyotype of Pyrrhulininae species with a high numeric and structural chromosomal variation are found in Lebiasinidae, thus differentiating the evolutionary pathways of both subfamilies.

The divergent evolutionary pathways between the genomes of Lebiasininae and Pyrrhulininae species are also demonstrated by our CGH experiments, where repetitive DNA sequences hybridized in different positions in their genomes, thus showing a high degree of genomic divergence among them. It is striking that divergent patterns of hybridization have occurred even among closely related species, such as *L. bimaculata* and *L. melanoguttata* [6], and *P. semifasciata* and *P. brevis* [5], revealed by species-specific CGH signals. In *Lebiasina*, this is a somewhat expected feature since *L. melanoguttata* is endemic, remaining isolated from distribution areas of several other lebiasinids by a distance of minimum 1500 km [37,38]. The presence of two other *Lebiasina* species (*L. marilynae* and *L. minuta*) in this same isolated area suggests the occurrence of allopatric speciation events [38], favoring the emergence of different patterns of genomic diversification. However, together with such general genomic divergence, it is also evident that inter-specific hybridization of repetitive sequences still occurs in *Lebiasina* chromosomes, in this way supporting the proposed basal position in the Lebiasinidae [16,17]. 

The distribution of ribosomal DNA sites is also a characteristic that experienced an extensive differentiation during the chromosomal evolution of Lebiasinidae species. Our review demonstrates that these sequences are distributed from a single site in the karyotype (i.e., *Lebiasina bimaculata*) to multiple ones (i.e., *Pyrrhulina australis*) and in a broad range of chromosomal locations. The evolution of rDNA sequences follows the concept of concerted evolution, maintaining the functionality and homogeneity of these genes [39,40]. However, since homologous and non-homologous recombinations are processes that mediate the concerted evolution, unequal sister chromatid recombination or retrotransposition may lead to favor a copy number variation of such sequences [41,42,43,44]. Indeed, this copy number variation can generate some non-transcribed rDNA copies that have extreme importance on genome integrity [45]. In fishes, copy number variation of ribosomal DNAs was extensively reported, since their gene regulation processes seem to be more relaxed than in higher vertebrates [42]. In turn, it is meaningful that Ctenoluciidae fishes possess a conserved pattern of rDNA distribution since, in this family, a single site of 18S rDNA is found in all species [4]. Therefore, as the basal genus *Lebiasina* shares this same pattern, this characteristic may have arisen before the split of Lebiasinidae and Ctenoluciidae.

The terminal position of the 18S rDNA in chromosomes appears common for *Nannostomus*, *Pyrrhulina*, *Lebiasina,* and *Copeina*. With the same pattern in the sister family Ctenoluciidae, this trait can be considered as symplesiomorphic [24]. The terminal position of 45S rDNA is a common characteristic for several groups, including fish, in contrast to the 5S loci that appear to have a more frequent interstitial location along the chromosomes [43]. However, this later condition of 5S rDNA does not apply to Lebiasinidae and even Ctenoluciidae, where both terminal and interstitial positions are observed, but with a preferential location at the chromosome termini in *Nannostomus* and *Pyrrhulina* chromosomes [3,4,5,24].

It is also noteworthy that genomes of *Nannostomus unifasciatus* and *Pyrrhulina brevis* exhibit particular arrangements of ribosomal DNAs. To some extent, this is an expected trait for *N. unifasciatus*, since this species has the lowest diploid number among lebiasinid fishes, with 2n = 22 and the karyotype formed by Robertsonian fusions [46]. In turn, peri- and/or paracentric inversions appear to have had an important role in the karyotype differentiation of *P. brevis* [5]. In this sense, besides the action of possible transposable elements, rDNA sequences may have been shifted by such rearrangements during the karyotype evolution. Furthermore, syntenic 5S and 18S sites in *Lebiasina melanoguttata* (pair 01), *P. australis* (pairs 07 and 14), *Pyrrhulina* aff. *australis* (pair 07), and *P. brevis* (pairs 03 and 14) were detected, and this situation may increase the recombination frequency [43], and, in association with heterochromatin, may act as recombination hotspots [47,48,49].

The evolutionary process may be highly influenced by chromosome rearrangements since they might facilitate the creation or the break of linkage-groups and alter gene expression [50,51]. Additionally, mechanisms for post-zygotic reproductive isolation may also be generated by chromosome fusions, for example [52]. It is also noteworthy that the distribution of repetitive DNA sequences could explain the genome dynamics from a chromosomal point of view, helping to untangle taxonomic issues [33,53,54], patterns of sex chromosome differentiation [5,21,22] and even recognizing hybridization events [55,56]. By that, in an evolutionary context, it is relevant that cytogenetical studies deliver chromosomal data for repetitive DNA distribution and chromosome rearrangements.

## 5. Conclusions

The studies of Arcila et al. [16], and Betancur-R et al. [17], indicate the proximity of the Lebiasinidae and Ctenoluciidae families, besides corroborating the monophyly of the two lebiasinid subfamilies, Lebiasininae and Pyrrhulininae. This means conventional and molecular cytogenetic data, which have been progressively improved for miniature fishes, actually corroborate and strengthen the proposed proximity relationship between Lebiasinidae and Ctenoluciidae. Additionally, it is also notorious as the evolutionary divergence that appears to differentiate both Lebiasinidae subfamilies. The chromosomal diversity in Pyrrhulininae hugely contrasts with the apparent conservatism of Lebiasininae. Furthermore, in addition to the specific repetitive DNA content that characterizes the genome of each particular species, *Lebiasina* also keeps inter-specific repetitive sequences, thus reinforcing its proposed basal condition within Lebiasinidae. The results now available provide significant advances in understanding the chromosomal evolution of Lebiasinidae fishes, a historically neglected group of the Neotropical Ichthyofauna in resolute cytogenetic investigations.

## Figures and Tables

**Table 1:** Collection sites and sample sizes (*N*) of the species examined. All from Brazil.

Species	Locality	*N*	Deposit Number
*Copeina guttata* Steindachner, 1876	Tefé, Amazonas (S03°23′07.7′′, W64°46′43.7′′)	11♀; 06♂	MZUSP 124915
*Copella nattereri* Steindachner, 1876	Manaus, Amazonas(S02°35′42.9′′, W60°02′23.8′′)	04♀; 06♂	MZUSP 124923
*Lebiasina melanoguttata* Netto-Ferreira, 2012	Cachoeira da Serra, Pará (S08°58′18,7′′, W54°58′18,7′′)	22♀; 14♂	MZUSP 124457
*Nannostomus eques* Steindachner,1876	Manaus, Amazonas (S02°47′58.1′′, W60°29′19.8′′)	02♀; 02♂	MZUSP 123084
*Nannostomus marginatus* Eigenmann, 1909	Manaus, Amazonas (S02°55′53.9′′, W59°58′30.7′′)	03♀; 05♂	MZUSP 123079
*Nannostomus beckfordi* Günther, 1872	Manaus, Amazonas (S02°55′53.9′′, W59°58′30.7′′)	09♀; 17♂	MZUSP 123071
*Nannostomus trifasciatus* Steindachner, 1876	Manaus, Amazonas (S02°44′59.6′′, W60°01′37.9′′)	07♀; 12♂	MZUSP 123071
*Nannostomus unifasciatus* Steindachner, 1876	Manaus, Amazonas (S02°47′58.1′′, W60°29′19.8′′)	05♀; 07♂	MZUSP 123083
*Pyrrhulina australis* Eigenmann & Kennedy, 1903	Santo Afonso, Mato Grosso (S14°27′25.2′′, W57°34′35.2′′)	30♀; 18♂	MZUSP 119079
*Pyrrulina aff. australis*	Barra do Bugres, Mato Grosso (S15°04′27.5′′, W57°11′05.4′′)	22♀; 16♂	MZUSP 119077
*Pyrrulina brevis* Steindachner, 1876	Manaus, Amazonas (S02°55′53.9′′, W59°58′30.7′′)	13♀; 17♂	MZUSP 124916
*Pyrrulina semifasciata* Steindachner, 1876	Tefé, Amazonas (S3°39′45.8′′, W64°35′33.3′′)	07♀; 12♂	MZUSP 123073
